# Effects of online-bandwidth visual feedback on unilateral force control capabilities

**DOI:** 10.1371/journal.pone.0238367

**Published:** 2020-09-17

**Authors:** Joon Ho Lee, Nyeonju Kang

**Affiliations:** 1 Department of Human Movement Science, Incheon National University, Incheon, South Korea; 2 Neuromechanical Rehabilitation Research Laboratory, Incheon National University, Incheon, South Korea; 3 Division of Sport Science & Sport Science Institute, Incheon National University, Incheon, South Korea; Federation University Australia, AUSTRALIA

## Abstract

**Purpose:**

The purpose of this study was to examine how different threshold ranges of online-bandwidth visual feedback influence unilateral force control capabilities in healthy young women.

**Methods:**

Twenty-five right-handed young women (mean±standard deviation age = 23.6±1.5 years) participated in this study. Participants unilaterally executed hand-grip force control tasks with their dominant and non-dominant hands, respectively. Each participant completed four experimental blocks in a different order of block presentation for each hand condition: (a) 10% of maximum voluntary contraction (MVC) with ±5% bandwidth threshold range (BTR), (b) 10% of MVC with ±10% BTR, (c) 40% of MVC with ±5% BTR, and (d) 40% of MVC with ±10% BTR. Outcome measures on force control capabilities included: (a) force accuracy, (b) force variability, (c) force regularity, and (d) the number of times and duration out of BTR.

**Results:**

The non-dominant hand showed significant improvements in force control capabilities, as indicated by higher force accuracy, less force variability, and decreased force regularity from ±10% BTR to ±5% BTR during higher targeted force level task. For both hands, the number of times and duration out of BTR increased from ±10% BTR to ±5% BTR.

**Conclusions:**

The current findings suggested that the narrow threshold range of online-bandwidth visual feedback effectively revealed transient improvements in unilateral isometric force control capabilities during higher targeted force level tasks.

## Introduction

Visual feedback is one of the effective modalities improving motor control functions [1, 2]. For example, while operating driving simulators, a driver with additional virtual driving lanes with error information can improve task performances [3, 4]. Presumably, descending commands corrected by the visual information during the actual action may influence on motor neuron pool activations contributing to advanced motor outcomes [5]. In fact, many researchers evidenced that an ability to successfully process the online-visual feedback, as referred to visuomotor processing function is crucial for enhancing fine motor control [5–8]. Isometric force control paradigm is useful for estimating visuomotor processing function by investigating the effects of various visual feedback types on altered force control capabilities [5, 7]. The accumulative findings indicated that the online-visuomotor corrections during isometric force control tasks were highly related to force fluctuations below 4 Hz [9–12]. Thus, administering isometric force control tasks may effectively contribute to understanding how the specific modulation of visual information affects motor outputs.

Two conventional modes of online-visual feedback during isometric force control tasks involve: (a) targeted force levels (i.e., task goals) and (b) isometric force production (i.e., the amount of force outputs generated by a performer) [2, 13]. Previous force control studies suggested a proposition that the greater amount of online-visual feedback (e.g., increased visual gain and higher frequency of visual information) may improve motor control capabilities in individuals with intact visuomotor processing functions [14–17]. For example, healthy young adults enhanced their isometric force control capabilities with online-visual feedback including both targeted force levels and isometric force production in comparison to those with only targeted force levels [18]. Moreover, when visual feedback on both targeted force levels and isometric force production were available during the tasks, high visual gains on force outputs additionally reduced force variability as compared with low visual gains [14]. Taken together, these findings indicated that modulating the amount of online-visual feedback may further advance visuomotor processing ability contributing to motor control capabilities.

Providing bandwidth visual feedback in addition to two traditional visual information (i.e., targeted force level and isometric force production) may facilitate more improvements in force control capabilities. The bandwidth visual feedback is a visual cue indicating that the motor performance is out of a predefined tolerance range such as overshooting upper limit of threshold and undershooting lower limit of threshold [19]. Several early studies provided bandwidth visual feedback in offline mode as a type of knowledge of results after each trial with a short task interval (e.g., force production within 1 s), and identified more accurate and less variable ballistic force outputs across trials [20–22]. Further, applying the offline-bandwidth visual feedback with a narrow threshold range (±5% of a targeted force level) showed more transient reduction of force variability across trials than those with a wide threshold range (±10% of a targeted force level) [20]. Perhaps, these short-term improvements were attributed to better motor programming adjustments facilitated by the offline visuomotor processing with a certain level of bandwidth threshold limits [23]. Moreover, while maintaining continuous force outputs around a target level during relatively longer trial intervals (e.g., 8–20 s), providing online-bandwidth visual feedback effectively decreased task error and variability of isometric force production as compared with no online-bandwidth visual feedback conditions [18, 24]. However, given that these findings were based on a fixed range of threshold limits for the bandwidth visual feedback, how altered threshold ranges of online-bandwidth visual feedback differently influence on force control capabilities is still unclear.

The purpose of this study was to investigate the effects of altered threshold ranges of online-bandwidth visual feedback on unilateral force control capabilities. For each hand condition (i.e., dominant and non-dominant), we compared two specific threshold ranges of the online-bandwidth visual feedback: (a) ±5% of a targeted force level (i.e., a narrow threshold range) versus (b) ±10% of a targeted force level (i.e., a wide threshold range). Moreover, we administered different targeted force level conditions (i.e., 10 and 40% of maximum voluntary contraction: MVC). Consistent with a prior finding that narrower threshold range of offline-bandwidth visual feedback transiently reduced force variability across trials [20], positive effects of the online-bandwidth visual feedback on unilateral force control capabilities would increase in a narrow threshold range condition. Moreover, right hemisphere controlling non-dominant hand may contribute to simultaneously stabilizing unstable motor outputs depending on the impedance control [25–27], and the non-dominant hand may require more visuomotor integration while producing and maintaining greater isometric force outputs using the online-visual feedback [28]. Thus, we hypothesized that a narrow threshold range of online-bandwidth visual feedback would improve force control capabilities in the non-dominant hand at higher targeted force level.

## Materials and methods

### Participants

Twenty-five young women (mean±standard deviation age = 23.6±1.5 years) with no musculoskeletal deficits or neurological disorders in their upper extremities participated in this study. All participants were right-handed healthy individuals based on the findings from the Edinburgh handedness inventory [29]. Specific details on demographic information are shown in Table 1. The Institutional Review Board at the Incheon National University approved the study protocols, and all participants read and signed an informed consent before participating in this study.

**Table 1:** Demographics of the participants.

Characteristics	Participants
Sample Size (N)	25 females
Age (years)	23.6±1.5
Handedness	25 right-handed
Weight (kg)	59.6±5.8
Skeletal Muscle Mass (kg)	23.7±2.2
Body Fat Mass (kg)	16.9±4.2
BMI (%)	22.2±2.5
Cognitive Functions (MMSE)	28.8±1.2

Data were mean±standard deviation. BMI: body mass index; MMSE: mini mental state examination [30].

### Experimental setup

**Fig 1:**
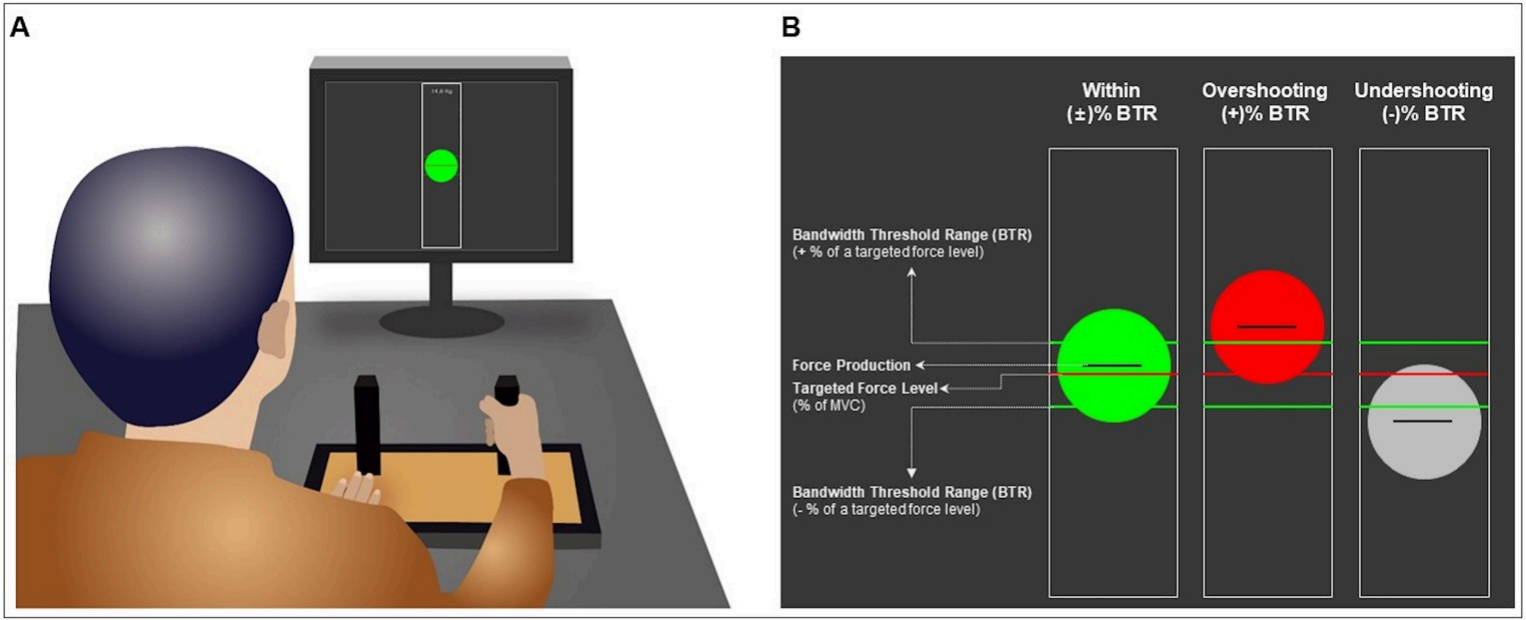
Experimental setup. (A) Participants used isometric hand-grip force measurement system to maintain their unilateral force outputs across a predefined threshold range including ±5 and 10% BTR conditions on the monitor. (B) Three types of online-visual feedback: (1) isometric force production: a black horizontal line in a circle, (2) a targeted force level: a red horizontal line on the center, and (3) upper and lower limits of BTR: two green horizontal lines. When the force outputs (i.e., a black horizontal line in a circle) stay within the upper and lower limits of BTR, a circle turns into green. When the force outputs (i.e., a black horizontal line in a circle) overshoot an upper limit (i.e., an upper green horizontal line) of BTR, the green circle turns into red. When the force outputs (i.e., a black horizontal line in a circle) undershoot a lower limit (i.e., a lower green horizontal line) of BTR, the green circle turns into gray. The size of circle was constant across all different experimental conditions, and the distance between two green horizontal lines was altered based on the level of BTR.

All participants unimanually performed isometric hand-grip force control tasks. Before the task execution, the participants sat 80 cm away from a 54.6 cm LED monitor (1920 × 1080 pixels; refresh rate = 60 Hz) and placed their both arms on the table with comfortable positions (15–20° of shoulder flexion and 20–45° of elbow flexion). We used the isometric hand-grip force measurement system (SEED TECH Co., Ltd., Bucheon, South Korea; Fig 1A) including left and right handles (a diameter = 30 mm) embedded with two connected force transducers, respectively (Micro Load Cell-CZL635-3135, range = 220 lbs, Phidgets Inc., Calgary, Canada). During the task execution with their unilateral testing hand, participants maintained to put their resting hand on the pad. Further, we instructed volunteers to fix both forearms down on the table to avoid any unintentional force production caused by elbow, shoulder, or trunk actions.

Initially, the participants completed three MVC trials (a trial interval = 5 s and 60 s rest between trials) with their dominant and non-dominant hands. We identified a maximum force for each MVC trial, and averaged the three peak force values. Based on these values, we selected 10 and 40% of MVC as targeted force levels for each hand condition.

During the isometric force control tasks, the participants tried to maintain their unilateral force outputs (i.e., black line in a green circle with a diameter = 46 mm) around the targeted force level (i.e., red line) for 30 s. While performing the tasks, we provided two types of online-bandwidth visual feedback: (a) overshooting an upper limit of threshold: turning a green circle to a red circle and (b) undershooting a lower limit of threshold: turning a green circle to a gray circle (Fig 1B). We used two specific threshold ranges of the online-bandwidth visual feedback: (a) ±5% of a targeted force level versus (b) ±10% of a targeted force level. A constant visual angle (= 1°) were maintained across all conditions [31].

Based on the prior findings [20, 22, 28], each participants completed four experimental blocks in a different order of block presentation for each hand condition: (a) 10% of MVC with ±5% bandwidth threshold range (BTR), (b) 10% of MVC with ±10% BTR, (c) 40% of MVC with ±5% BTR, and (d) 40% of MVC with ±10% BTR. Given that each block consisted of three consecutive trials, participants completed 12 total trials with their dominant and non-dominant hands, respectively. To minimize the involvement of muscle fatigues, we provided a 30 s of rest period between trials and a 60 s rest period between blocks.

All force data were sampled at the rate of 100 Hz using a 16-bit analog-to-digital converter (A/D; ADS1148 16-Bit 2kSPS and a minimum detectable force = 0.0192 N), and amplified using an INA122 with an excitation voltage of 5 V (Texas Instruments Inc., Dallas, USA). We used a custom Microsoft Visual C++ Program (Microsoft Corp., Redmond, USA) for administering the experimental procedures, and applied a custom Matlab Program (Math Works^™^ Inc., Natick, USA) for offline analyses.

### Data analyses

For 30 s of each trial, we used the middle 20 s of force data after removing the first 5 s and the final 5 s to minimize potential early initial adjustment and termination effects. We filtered the trimmed force data using a bidirectional fourth-order Butterworth filter at 30 Hz of cut off frequency. To estimate force control capabilities, we calculated following outcome measures: (a) force accuracy: root-mean-square error (RMSE), (b) force variability: coefficient of variation (%CV) = *SD* of force / mean force × 100, and (c) force regularity: approximate entropy (ApEn; Formula 1) [32–34], and (d) the number of times and duration (second) out of BTR. The ApEn values close to zero indicate more regular force outputs, whereas the ApEn values close to two denote more irregular force outputs [35, 36].

$$ApEn(\vec{X},m,r)=In\;\left[\frac{C_{m}(r)}{C_{m+1}(r)}\right]$$ (1)

where *C_m_*(*r*) means prevalence or repetitive patterns of length *m* in vector *X* (i.e., force date in time samples). *m* (= 2) is specific pattern length and *r* (= 0.2 × *SD*) is criterion of similarity [35, 37, 38]. In addition, we calculated the number of times and duration out of either upper or lower limits of threshold.

For the statistical analyses, we performed two-way repeated measures ANOVA (Threshold Range × Force Level; 2 × 2) on the dependent variables for each hand condition. We confirmed the normality of all dependent variables using the Shapiro-Wilk’s W test [39, 40]. For post hoc procedures, we used Bonferroni’s pair-wise comparisons. All statistical analyses were conducted using the IBM SPSS Statistics 22 (SPSS Inc., Chicago, IL, USA) and we set an alpha level at 0.05.

## Results

### Force accuracy: RMSE

**Fig 2:**
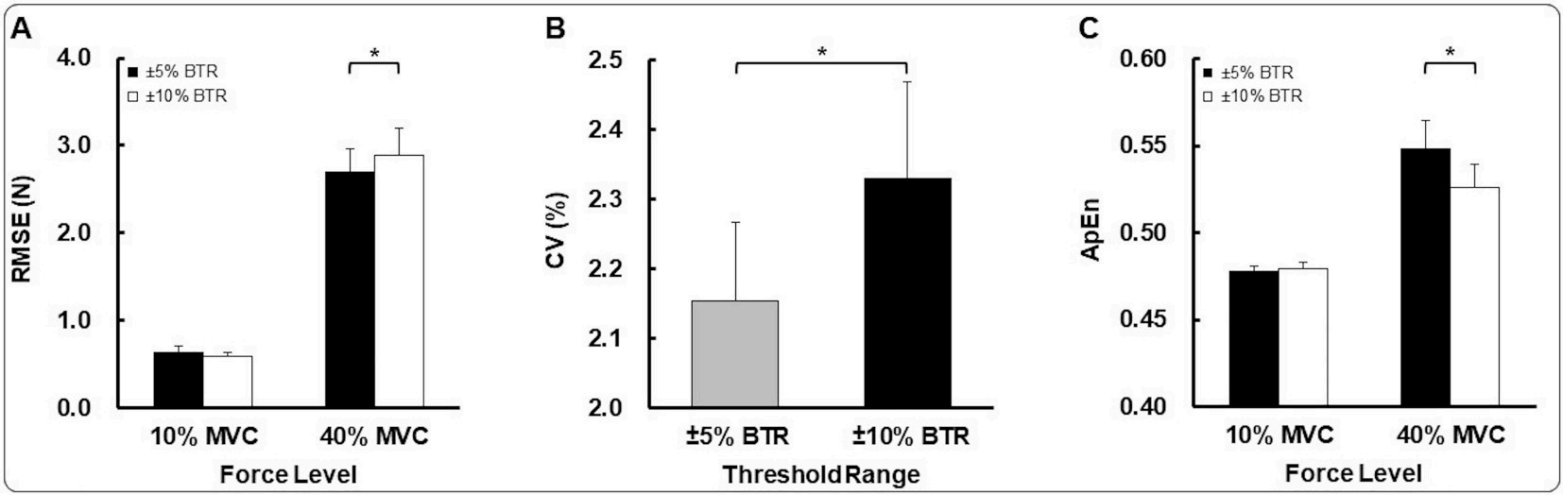
Unilateral force control for non-dominant hand condition (*M*±*SE*). (A) Force accuracy (RMSE) for BTR conditions (±5 and 10% BTR) as a function of force levels (10 and 40% of MVC). (B) Force variability (CV) for BTR conditions (±5 and 10% BTR). (C) Force regularity (ApEn) for BTR conditions (±5 and 10% BTR) as a functions of force levels (10 and 40% of MVC). BTR means a predefined threshold range of online-bandwidth visual feedback. *Asterisk* (*) indicates a significant difference between variables. *P* < 0.05.

For the non-dominant hand, the two-way repeated measures ANOVA on RMSE revealed a significant Threshold Range × Force Level interaction [*F*(1,24) = 4.744; *P* = 0.039; η^2^ = 0.165; Fig 2A]. Post hoc analysis showed that RMSE values at 40% of MVC were significantly lower in ±5% BTR than ±10% BTR (*P* = 0.047). These findings indicate that the narrow threshold range of online-bandwidth visual feedback improved force accuracy while executing unilateral non-dominant hand motor control at the higher targeted force level.

**Fig 3:**
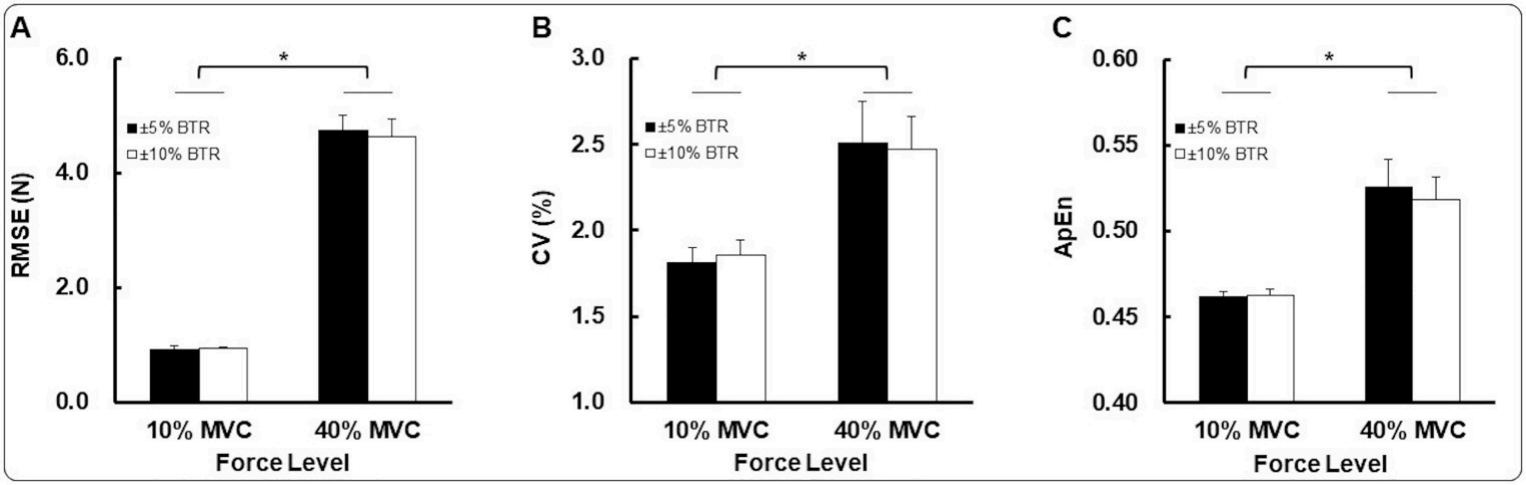
Unilateral force control for dominant hand condition (*M*±*SE*). (A) Force accuracy (RMSE) for BTR conditions (±5 and 10% BTR) as a function of force levels (10 and 40% of MVC). (B) Force variability (CV) for BTR conditions (±5 and 10% BTR) as a function of force levels (10 and 40% of MVC). (C) Force regularity (ApEn) for BTR conditions (±5 and 10% BTR) as a functions of force levels (10 and 40% of MVC). BTR means a predefined threshold range of online-bandwidth visual feedback. *Asterisk* (*) indicates a significant difference between variables. *P* < 0.05.

For the dominant hand, the analysis revealed a significant force level main effect [*F*(1,24) = 20.921; *P* < 0.001; η^2^ = 0.466; Fig 3A]. Specifically, RMSE values were significantly lower at 10% of MVC than 40% of MVC (*M*±*SE*): (a) 10% of MVC = 0.9±0.3 N and (b) 40% of MVC = 4.7±1.1 N. These findings indicate that unilateral dominant hand reduced task error during isometric force control at the lower targeted force level.

### Force variability: CV

For the non-dominant hand, the two-way repeated measures ANOVA on CV showed a significant threshold range main effect [*F*(1,24) = 8.572; *P* = 0.007; η^2^ = 0.263; Fig 2B]. Specifically, CV values were significantly less in ±5% BTR than ±10% BTR (*P* = 0.007). These findings indicate that the narrow threshold range of online-bandwidth visual feedback decreased force variability produced by unilateral non-dominant hand collapsed across two different targeted force levels.

For the dominant hand, the analysis showed a significant force level main effect [*F*(1,24) = 8.996; *P* = 0.006; η^2^ = 0.273; Fig 3B]. CV values were significantly lower at 10% of MVC than 40% of MVC (*M*±*SE*): (a) 10% of MVC = 1.84±0.08% and (b) 40% of MVC = 2.49±0.21%. These findings indicate that unilateral dominant hand decreased force variability at the lower targeted force level.

### Force regularity: ApEn

For the non-dominant hand, the two-way repeated ANOVA on ApEn revealed a significant Threshold Range × Force Level interaction [*F*(1,24) = 5.016; *P* = 0.035; η^2^ = 0.173; Fig 2C]. Post hoc analysis revealed that ApEn values at 40% of MVC significantly increased from ±10% BTR to ±5% BTR (*P* = 0.024). These findings indicate that the narrow threshold range of online-bandwidth visual feedback reduced force regularity while executing unilateral non-dominant hand motor control at the higher targeted force level.

For the dominant hand, the analysis showed a significant force level main effect [*F*(1,24) = 18.368; *P* < 0.001; η^2^ = 0.434; Fig 3C]. ApEn values were significantly higher at 40% of MVC than 10% of MVC (*M*±*SE*): (a) 10% of MVC = 0.46±0.01 and (b) 40% of MVC = 0.52±0.02; *P* < 0.001. These findings indicate that force regularity produced by unilateral dominant hand decreased at the higher targeted force level.

### The number of times and duration out of BTR

For the non-dominant hand, the two-way repeated ANOVA on the number of times out of BTR revealed a significant threshold range main effect [*F*(1,24) = 23.021; *P* < 0.001; η^2^ = 0.490]: (a) ±5% BTR (*M*±*SE*) = 16.8±2.1 and (b) ±10% BTR (*M*±*SE*) = 3.5±1.7; *P* < 0.001. Additionally, the analysis on the duration out of BTR revealed a significant threshold range main effect [*F*(1,24) = 24.907; *P* < 0.001; η^2^ = 0.509]: (a) ±5% BTR (*M*±*SE*) = 2.6±0.4 s and (b) ±10% BTR (*M*±*SE*) = 0.4±0.2 s; *P* < 0.001. These findings indicate that the narrow threshold range of online-bandwidth visual feedback increased the number of times and duration while executing unilateral non-dominant hand motor control.

For the dominant hand, the two-way repeated ANOVA on the number of times showed significant threshold range and force level main effects: (a) threshold [*F*(1,24) = 36.330; *P* < 0.001; η^2^ = 0.602] and (b) force level [*F*(1,24) = 7.115; *P* = 0.013; η^2^ = 0.229]. The number of times out of BTR was higher in ±5% BTR than ±10% BTR (*M*±*SE*): (a) ±5% BTR = 11.7±1.6 and (b) ±10% BTR = 1.4±0.4. The number of times out of BTR was greater at 40% of MVC than 10% of MVC (*M*±*SE*): (a) 40% of MVC = 8.4±1.3 and (b) 10% of MVC = 4.7±0.9. Moreover, the analysis on the duration out of BTR revealed a significant Threshold Range × Force Level interaction [*F*(1,24) = 9.580; *P* = 0.005; η^2^ = 0.285]. Post hoc analysis revealed that increased duration out of BTR appeared at 40% of MVC from ±10% BTR to ±5% BTR (*M*±*SE*): (a) ±5% BTR = 2.9±0.8 s and (b) ±10% BTR = 0.8±0.6 s; *P*<0.001. These findings indicate that the narrow threshold range of online-bandwidth visual feedback increased the number of times and duration out of BTR while performing unilateral dominant hand motor control.

## Discussion

We investigated the effects of altered threshold range of online-bandwidth visual feedback (±5% versus ±10% BTR) on unilateral isometric force control in healthy young women. The non-dominant hand significantly improved force control capabilities in the narrow BTR condition at the higher targeted force level (i.e., 40% of MVC), as indicated by higher force accuracy, less force variability, and decreased force regularity. However, altered threshold range of the online-bandwidth visual feedback did not influence the dominant hand’s force control capabilities. For both hands, the number of times and duration out of BTR increased in the narrow BTR condition.

In addition to the two conventional visual feedback including targeted force level (i.e., task goal) and isometric force production generated by a performer, providing concurrent bandwidth feedback with ±5% BTR showed higher force accuracy in the non-dominant hand than those with the online-bandwidth visual feedback in ±10% BTR. These findings were consistent with prior studies that used bandwidth visual feedback after completing feedforward control of force production within a relatively short task interval (i.e., task duration = 200–1,300 ms for each trial) [20–22]. Specifically, the offline-bandwidth visual feedback with a predefined tolerance range improved force accuracy across multiple trials, and these transient improvements were greater in the narrower predefined tolerance range (i.e., ±5% BTR) than ±10% BTR. Further, several studies examined the effects of online-bandwidth visual feedback on force control with relatively longer task intervals [3, 18, 24], and found more reduction of task error [3, 24]. Thus, our findings expanded these results by showing that the narrower predefined tolerance range of online-bandwidth visual feedback may be more effective to advance force accuracy.

Moreover, the online-bandwidth visual feedback with ±5% BTR reduced force variability in the non-dominant hand as compared with ±10% BTR. In a previous study when a predefined tolerance range of the offline-bandwidth visual feedback was relatively narrower (i.e., ±5% BTR), participants successfully reduced variability of motor outputs [20]. Beyond the effects of offline-bandwidth visual feedback, we additionally confirmed that providing the online-bandwidth visual feedback with narrower BTR effectively minimized inconsistency of motor outputs during task execution. Greater amount of visual information (e.g., increased visual gain) may decrease motor variability during continuous isometric force production tasks [41, 42] because of the reduced neural noise (e.g., synaptic noise of motor neurons pool) on motor unit discharge [43]. Similarly, given that the narrow BTR of bandwidth visual feedback may provide more frequent visual cues to a performer during isometric force control tasks [20], the increased amount of visual information presumably minimized neural noise contributing to less force variability.

The narrow BTR of online-bandwidth visual feedback improved individual’s motor adaptability as indicated by less force regularity. These findings may support an entropy compensation assumption in human motor behaviors indicating the compensatory tradeoff between both environmental entropy and motor entropy [44]. For example, when environmental information increases (i.e., less environmental entropy) during the motor task, motor regularity decreases (i.e., higher motor entropy). In fact, previous findings indicated that an increase in either visual gain or frequency of visual feedback on force outputs improved temporal structure of force variability [16, 45, 46]. Presumably, the narrow BTR of online-bandwidth feedback reduced the environmental entropy so that individuals may increase their motor entropy as compensatory actions.

While executing unilateral isometric force control tasks, the number of times and duration out of BTR were greater in the narrow BTR of online-bandwidth visual feedback than those in the wide BTR for both hands. However, these results did not negatively affect force control capabilities in the non-dominant hand, as indicated by more improvements in force accuracy, variability, and regularity in the narrow BTR condition. Similarly, the dominant hand revealed no differences in force control capabilities between two BTR conditions. Perhaps, these findings indicated that more frequent appearance of online-bandwidth visual feedback may not compromise online-motor corrections in the motor system.

According to the motor lateralization model [27], two hands may have different neuromuscular functions based on the hemispheric functional specialization. For example, the left hemisphere controlling the dominant hand contributed to accurately generating predictive dynamic motor outputs. In contrast, the right hemisphere controlling the non-dominant hand was related to an ability to simultaneously stabilize unstable motor outputs using impedance control (e.g., a reactive force production to deal with unpredictable external perturbations) [25–27, 47]. These findings raised a possibility that the non-dominant hand may facilitate to stabilize unstable forces in response to more frequent appearance of online-bandwidth feedback (i.e., greater number of times and duration out of BTR) in the narrow BTR condition. Interestingly, brain activation patterns related to visuomotor processing were different between the dominant and non-dominant hand execution conditions [28]. While performing isometric grip force control tasks with unilateral non-dominant hand, the sensorimotor network including the cerebellum, supplementary motor areas, and right posterior parietal cortex was highly activated at a higher targeted force level. However, these patterns were not observed in the unilateral dominant hand condition. At the higher targeted force level, the non-dominant hand may be more dependent on additional neuronal resources from the sensorimotor network for successful sensory recalibration and motor adaptation [48, 49]. Taken together, these findings suggested that the frequent appearance of online-bandwidth visual feedback in the narrow BTR condition may facilitate the visuomotor integration contributing to improved force control capabilities in the non-dominant hand.

Despite the potential effects of the narrower online-bandwidth visual feedback on non-dominant hand force control, we need to consider some limitations in this study. First, given that only healthy young women participated in this study, our findings may not extend to other population such as males and older adults who presumably have different prior experiences on unimanual isometric grip force control. Second, we used an isometric force control paradigm so that positive effects of the online-bandwidth visual feedback on dynamic force control capabilities, highly related to various functional movements are still uncertain. Thus, future studies should investigate how the online-bandwidth visual feedback effects various force control functions (e.g., isometric and dynamic tasks) in healthy men and aging groups.

## Conclusions

The current study revealed that providing online-bandwidth visual feedback from ±10% BTR to ±5% BTR significantly improved force control capabilities, as indicated by higher force accuracy, less force variability, and decreased force regularity. Specifically, these transient improvements appeared when individuals performed relatively at the higher targeted force level with their non-dominant hand. These findings suggest that the non-dominant hand in young women may show more dependence on the extrinsic feedback augmented by the narrow threshold range of online-bandwidth visual information because of the different neuromuscular functions between hands.
